# The synergistic relationship between Amartya Sen entitlement theory and the systems theory in developing a food security implementation model in Matabeleland South Province, Zimbabwe

**DOI:** 10.4102/jamba.v13i1.965

**Published:** 2021-04-08

**Authors:** Tapiwa Muzerengi, Ernest N. Khalema, Emmanuel Zivenge

**Affiliations:** 1 Department of Agricultural Economics, Education and Extension, Faculty of Agriculture and Environmental Science, Bindura University of Science Education, Bindura, Zimbabwe; 2 School of Built Environment and Development Studies, College of Humanities, University of KwaZulu-Natal, Durban, South Africa

**Keywords:** availability, accessibility, systems, entitlement, community

## Abstract

Food insecurity has increasingly become a topical issue that needs to be addressed before it goes out of hand. The article explores the synergistic relationship that exists between Amartya Sen entitlement and systems theories. The article hypothesises that food insecurity in Matabeleland South Province is mainly caused by a lack of understanding of food security pillars and how all the concepts dove tail into the food security discourse. The article further propounds that, for communities to go out of the food insecurity quagmire, they need to work collaboratively as a system as substantiated by the systems theory. Sen argues that the law stands between food availability and access. This is further simplified as follows: food can be available in the markets but the people might lack the purchasing power to purchase the food. When people try to acquire food through stealing, the law catches on them. On the other hand, the systems theory argues that for a community to function well all the parts should play their roles towards the survival of the whole. The theory further informs that government stakeholders and non-state actors must work together in addressing food insecurity without a clearly defined direction. Interactions with provincial stakeholders, revealed that, a leaf can be borrowed and applied from the two theoretical models to achieve food security in Matabeleland South Province in Zimbabwe.

## Introduction

This research article is hinged on Sen’s entitlement theory and the systems theory. The two theories complement each other and perfectly address the study thrust. Food security is a human entitlement, which can be achieved through ensuring stable and sustainable availability, accessibility and utilisation of food. This is the major thrust of Sen’s entitlement theory when it views entitlements as a set of commodity bundles that can be converted to address food security in a household. Systems comprising both human beings and other necessary resources and structures, should be developed because they feed into each other to promote synergies for sustainable food security in a given community. The entitlement theory looks at how communities can become food secure specifically looking at food access; whilst the systems theory looks at how the communities can become food secure. A combination of the two theories helps the study to answer all the research questions as they play a complementary role. This research took into consideration this vital need to develop a food security implementation model for Matabeleland South Province and this will go a long way in ameliorating food shortages in the province. Matabeleland South Province was chosen because it is in agroecological region five, which is characterised by early termination of rains, high temperatures and communities that are vulnerable and food insecure. To address the food insecurity quagmire, it was necessary to develop a context-specific food security implementation model. The two theories worked hand in glove to answer the research objectives of the study.

## Amartya Sen entitlement theory

Sen’s entitlement theory is one of the theories underpinning and influencing this study; the basis of this theory came from the analysis of famines. Entitlements have been defined by Sen (1984:497) as ‘the set of alternative commodity bundles that a person can command in a society using the totality of rights and opportunities that he or she faces’. This would involve everything that a person has that can bring food on the table; it could be in the form of food or non-food material. Sen (1981:166) resolved famines and poverty with this well-known scrutiny: ‘The law stands between food availability and food entitlement’. This dove tails into interrogating the legislative and enforcement regulations on food imports.

## Legislation and food enforcement

In Zimbabwe, there are different statutes that are used by the government to regulate the importation of food from neighbouring countries such as South Africa and Botswana. Of late, the government of Zimbabwe introduced Statutory Instrument 64 of 2016, which prohibits the importation of foodstuffs from outside the country. When the President of the Republic of Zimbabwe declared food (in) security as a national disaster, only selected licensed businesses were allowed to source food from other countries. The government of South Africa on 17 November 2017, pronounced the regulations relating to miscellaneous additives in food stuffs. This has been enforced on both domestic and imported food stuffs (Food and Agricultural Import Regulations 2019). The legislative instruments specifically focused on foodstuffs intended for both infants and young children. These regulations greatly affected food availability in the neighbouring Zimbabwe as propounded by Sen on the negative relationship between the law and food availability.

However, approximately half a million individual people were prohibited to source food on their own (Zimbabwe Vulnerability Assessment Committee [ZIMVAC] 2016). This is a clear indication, as resonated by Sen, that the law stands as a barrier between food availability and food entitlement, that is, the law acts as a barrier that restricts people from getting food from the shops, farms and neighbours only to mention a few. If people try to obtain food from those who have it without their consent it might be regarded as a serious crime. In other words, it may mean that even though food is available, it might not be easy to access it because of either exorbitant prices or inaccessible roads leading to hunger and starvation of the general populace. Edkins (1996:550) propounded that, in ‘Sen’s model, people destitute by famine are not entitled to food; instead they are entitled to starve’. Matabeleland South Province is well known for erratic rainfall that leads to poor field harvests, which in turn lead to severe food shortages. Severe food shortages are a major problem and people starve almost every year. The individual’s right set is the whole variety of services and goods that she or he can obtain by changing her or his endowments, that is, possessions and income, as well as effort power. It can be argued that, although the people of Matabeleland are able to sell assets (their labour and other endowments), this cannot be regarded as a panacea to the question of food (in) security in the province. Hence, the province should embark on a coordinated implementation food (in) security model that ensures that food will be brought to the table for the people in the province.

This model further asserts that, in the event of hunger, people can sell their belongings to get money so that they can support their families. These families, however, can actually work and in return they can be given money that can be used to buy food, hence bringing food security in the family. In the context of poverty and famine, the entitlement approach aims comprehensively to describe all legal sources of food. Sen (1981:2) reduced these sources to four categories: ‘production-based entitlement, trade-based entitlement, own labour entitlement and inheritance and transfer entitlement’. The production-based entitlement focuses on growing food, for example, by using irrigation schemes. The trade-based entitlement looks at buying food from irrigated farm produce. The own labour-based entitlement deliberates on working for food in these irrigation schemes and in return people are rewarded. The inheritance and transfer entitlement look at food given by others when irrigated produce reach fruition and the ones who are in dire need of food can also benefit. It connotes that the family can also benefit from the deceased’s property to obtain food from insecurity bag. Persons encounter hunger when their complete right set does not give them sufficient food for survival.

People are in dire need of food, yet the government of Zimbabwe is implementing its economic blueprint: Zimbabwe Agenda for Sustainable Socio-Economic Transformation (ZimASSET) with its first cluster on improving food security and nutrition. There are signals indicating that there is little or no trade, no employment, no exchange of labour because production is at stand still and food crisis goes up.

Domesticating the entitlement theory to food (in) security in Matabeleland South Province is a reality because when food insecurity has nested in a village or area, entitlement exchange declines drastically. Normally when people are in abject poverty, the power to negotiate becomes less, just as an English proverb says: ‘beggars cannot be choosers’. This will lead to a sudden fall in both wages; livestock prices and food prices start to skyrocket. However, the sudden fall of all the said variables might be a result of an uncoordinated Food (in) Security Implementation Model. When the implementation Model is executed holistically, it would mean that, markets for different products would be known by almost everyone. This makes the entitlements to fetch a better price instead of selling livestock at a very low price. For Sen, it is ordinary and his followers to do away with antagonists of the entitlement approach as ‘misleading’, ‘misinterpreting’, or even ‘misrepresenting’ Sen’s intentions.

Sen (1981:62) resonated that the entitlement strategy is expressive rather than hypothetical and empiricist rather than normative, ‘a general model for analysing famines rather than one particular hypothesis about their causation’. It is very imperative to borrow the contents of this theory because it helps to define a phenomenon rather than prescribing it. When a phenomenon is clearly understood, it is very easy to come up with an intervention strategy. In this instance, it helps the Province to come up with an informed type of an implementation model, which addresses food (in) security in the Province of Matabeleland South.

Amartya Sen throughout has given both a universal logical model for investigating all famines (the entitlement approach) and simultaneously a ‘new’ hypothesis of causation that specific famines are described by decreases in access to food for identifiable populace groups regardless of available food at national level (‘exchange entitlement failure’). Food might be available at national level, for example, Grain Marketing Boards (GMBs), but at district level people might be starving because they are not able to access food from GMBs because of prices, distance or any other reasons. Hence, the ZimASSET implementation model will come into play to address specific causes as highlighted by Sen’s causation theory. It can be easy to address a problem if a root cause is known.

Sen’s entitlement theory basically approaches food security from access point of view. It further spells out the barriers such as the law that can stand between food availability and food access. The theory believes that food can be available but at the same time not accessible to everyone. This can be witnessed by the abundance of food in supermarkets in the province whilst people are food insecure. The theory helps to finish the food chain from the producer to the consumer. The theory further asserts that, in the event of famines, people can sell their belongings so that they can get food, although those without or in abject poverty can exchange their labour at a very low cost. However, all these imbalances in food insecurity can be addressed when a food insecurity implementation model that addresses food shortages in the province is mooted. The model is people-centred and it brings everyone’s concerns on board. This helps to improve the food security status in the province.

## The systems theory

The systems theory cuts across the study of systems. Gwirayi, (2012) propounded that a system is a unified collection of consistent and mutually dependent parts that are either artificial or natural. Each system is defined by its temporal and spatial boundaries, bordered and affected by its surroundings, defined by its purpose or nature and structure articulated in its performance. In conditions of its effects, a system is bigger than the sum of its parts if it expresses emergent behaviour and synergy. Altering a single part of the system usually has an impact on other parts and the entire system with expected traits of behaviour. The aim of the systems theory is to thoroughly discover a system’s constraints, conditions, dynamics and explaining fundamentals that can be applied and discerned to systems at each level of nesting and in every field for attaining heightened equifinality.

Von Bertalanffy views a system as, ‘elements in standing relationship’. A system is an organised entity made up of interrelated and interdependent parts. The development of an implementation model to address food shortages in the province of Matabeleland South Province of Zimbabwe also needs to be deeply rooted in the systems theory. The systems theory was propounded by Cristina Mele, Jacqueline Pels and Francesco Polese. The implementation of the food (in) security model does not happen in a vacuum but in a community with conscious human beings. These human beings have different religious faiths, culture and even geographical specificities. It is prudent, however, to take Matabeleland South Province as a system that has parts that are connected together towards the survival of a whole. Facts are realised from the deep appreciation of the entire and not that of the individual parts (Aristotle’s Holism); researchers were grappling with systems and parts in terms of their relative dynamics and contents (Gwirayi 2012). Based on Gwirayi’s assertion, it can be posited that it is imperative to understand how the system works so that it can be easy to synchronise different parts of the system so that they can be meaningful.

However, Gwirayi’s assertion did not fully explain the relationship between a system and its elements. It should be noted that, a system is made up of small parts that are interrelated, interact and work towards the survival of the whole system. Hence, it can be less meaningful for one to concentrate on the functioning of the whole system without observing the small parts in a particular system. This momentous effort emerged during the previous century into the so-called ‘systems theory’ (Bogdanov 1922; Meadows 2008; Von Bertalanffy 1968). This study continuously employs the systems theory because its tenants are of relevance in coming up with a sound implementation model that is community driven and at the same time addresses food (in) security in Matabeleland South Province of Zimbabwe.

A system can be referred to as an entity, which is a unified whole (Ng, Maull & Yip 2009:22) in such a way that a demarcation is apparent around it in order to differentiate external and internal components and to recognise output and input pertaining to and evolving from the unit. Von Bertalanffy (1956) defined a system as a complex of interacting elements. Looking closely at the two given definitions, it can be deduced that a system is characterised by its coherence and interrelatedness of parts. It can be further argued that, a system is complex in nature and has different elements, which are coordinated and intertwined to serve a single purpose. The systems theory further asserts that an element in a system cannot be bigger than a system but rather small enough to combine with other elements to strengthen the system. In simple terms, this system can be viewed as a unit, which is solid and contains small parts that interact together and can be summed up together to form one whole. It means, these parts depend on each other, no one part is independent of the other and they interact for a common purpose.

Von Bertalaffy encourages systems philosophy in all subjects in a view to find general values important to all systems (Checkland 1997). A fundamental notion of the general systems theory is its focus on interactions (Boulding 1956). The centre in relationships with other elements of the system lead to sustain that the behaviour of a single autonomous element is different from its behaviour when the element interacts with other elements (Espejo Villar 1999). A systems theory is, therefore, a theoretical model that analyses a problem seen as a whole and not as simply the total number of elementary parts. Matabeleland South Province can be equated to a system. This system can be viewed as a whole. Inside the system, there are small parts that can be called districts and wards. In these districts and wards, respectively, there are different stakeholders such as heads of government ministries, representatives of the non-governmental organisations, rural district councils, local leadership and the community members at large. The major trust is on the relationships and interactions between parts in a bid to appreciate a unit’s outcomes, functioning and organisation structure, functioning and outcomes (Ashby et al. 2000).

The major characteristic of a system is that there should be interaction amongst the parts. This theory is very useful in this study in the sense that for a sound implementation model to address food (in) security to be done, interaction is needed from the community members, local leadership and heads of different government ministries, local authority and even the rural district councils. When all these stakeholders are interacting, it means that they are working together towards the survival of a whole, which in this case is the Province of Matabeleland South. When every element of the province is taken on board that means every geographic space in the province, culture, religion, social and economic status of everyone is represented. The implementation model is owned by everyone. In other words, the systems theory dwells on home grown solution to a problem. The phenomenon of food (in) security is tormenting Zimbabwe in general and Matabeleland South Province in particular. Hence, the systems theory contextualises the problem per province and promotes the interaction of stakeholders to work together towards the accomplishment of a common goal.

A number of implementation models have collapsed because of two reasons: (1) the implementation was imposed from the top or, (2) it was imposed from the bottom but lacked interaction between stakeholders. This theoretical model advocates for a dialogue between holism and reductionism. When developing an implementation model to alleviate food (in) security in the Province of Matabeleland South it is imperative to treat each stakeholder with the respect that each deserves. Different contributions from stakeholders give the total number of contributions for the whole province. Hence, the role of researchers and developers should not be to impose the implementation model, rather the people of Matabeleland South Province should come up with their own product. The role of the researchers should be to facilitate the development of an implementation model. Moreover, the researcher should be part of the system, which also interacts with other parts so that the interaction is purposeful towards the establishment of a solid whole.

The systems theory is an interdisciplinary theory about every system in nature, in society and in many scientific domains and a model with which we can investigate phenomena from a holistic approach (Capra 1997). The systems theory cuts across all the disciplines, hence its employment in community development is of significance. It can be assumed that the problem of food (in) security should be viewed with a holistic eye. However, the execution of actions in addressing this problem should be shared amongst different parts of the province. Systems thinking come from the shift in attention from the part to the whole (Checkland 1997; Jackson 2003; Weinberg 2001), considering the observed reality as an integrated and interacting unicum of phenomena where the individual properties of the single parts become indistinct. When parts work independently without relationships, this can cause a serious discord and it is hard to form a system. In a similar manner, when the stakeholders in the province do not work together, it is very difficult to come up with a solid system, which can produce a shared implementation model that addresses food (in) security in the province. In contrast, the relationships between the parts themselves and the events they produce through their interaction become much more important, with the outcome of ‘system elements are rationally connected’ (Luhmann 1990) towards a shared purpose (Golinelli 2009). What is of paramount importance in the systems theory is the relationship between the parts and the result that is obtained from working together.

In other words, the systems theory is goal-oriented or guided by task accomplishment. Task accomplishment can only be achieved when the elements are strongly connected together with sense of unity. This is the same that is expected in the province of Matabeleland South for communities to be connected and unite to come up with a working implementation model that unpacks the prospects of the Agenda for Sustainable Socio-Economic Transformation, which would bring a practical solution to acute food shortages in the province. All this can be realised through interaction and unity of purpose amongst different interrelated parts in the province. A major characteristic of a system is that, it believes that every part is equally important and no part is better than the other. Each part contributes equally towards the survival of the whole system. When it comes to stakeholder participation in the province, no stakeholder is better than the other but each is of equal importance in the system.

The systems theory resonates that it is not possible to completely understand a problem simply by splitting it up into smaller parts and then remould it; we instead want to demonstrate a holistic view to underscore its usefulness. A system displays a true picture of a whole, which needs interaction amongst the parts. Even though we can begin from the scrutiny of the basic elements of a problem, in a view to wholesomely understand the problem in its fullness we need to scrutinise it also from an elevated level: a holistic perspective (Von Bertalanffy 1968). The previous assertion by Von indicates that, although it is commendable to analyse the elements of the system for the good functioning of the system, we should strike a balance between the analysis of elements and of the system itself. Hence, the problem of food (in) security should be viewed in a holistic manner.

In a nutshell, the major content of the systems theory is that a system is a whole, which has interrelated parts that work together towards the fruition of the whole system. The elements in a system should have relationships that are meaningful in nature. No one element in a system is bigger than the other but all work equally together to achieve a common goal. Although the analysis of elements is needed in a system, it is of paramount importance to have a holistic view of the system itself to understand the problems affecting the system. A systems theory can be equated to human pathology where different parts of the body have different functions but all work in unison towards the survival of the whole body. This can also be transferred to the Province of Matabeleland South situation: different parts of the body connected together, that is, the food security stakeholders working together to address the food (in) security issue in the province. These stakeholders might interact together at district level as rural district development committees and feed to form the provincial development committee with a shared view from the communities of developing an implementation model that addresses food (in) security, which is one of the major thrusts of the ZimASSET.

## Methodology and food security implementation model

The study is ingrained in a completely qualitative methodology so that the participants are able to give their views in a natural set up using the community as a laboratory. Purposive sampling was used for targeting the key informants, of which 14 participants were selected two each from the department of agriculture, the local authority, the provincial administrator’s office, department of social welfare, GMB, farmers union and traditional leadership. The proposed food security implementation model for Matabeleland South Province consists of four phases: (1) point of community engagement, (2) skills-resource screening and/planning, (3) implementation stage and (4) progress review stage. It is imperative to note that the proposed implementation is iterative in nature, that is, it is very flexible and allows implementers to proceed or revisit mistakes for the common good of the community in as far as improving food security is concerned.

The first step was community engagement, that is, the entry point where the local people or community first meet to interact with experts in the food security sector. This was a necessary stage where rapport is built on a more personal and grass root level. During the process of this study, it was established that most of the intervention programmes implemented at the community lacked participatory edge. At this stage, that is, where the community spells out aims and objectives, consultations are made with food security stakeholders, entry of experts where they will be interacting with communities, awareness and demystification of policies. For the purpose of this research and the objective of participatory process development, this is where aims and objectives of the intended programme are spelt and explained to communities. Communities need to be fully engaged at this particular phase.

The second step was the skills-resource screening and/planning. Finding the skills available in the province was very essential in order to find synergies and how the available skills can complement each other. The third step is implementation of the development of a model. It was at this stage whereby the communities affected by food insecurity recommended that there should be community driven food security intervention strategies. The last stage was that of progress review. At this stage, monitoring and evaluation is done at ward level, district level and provincial level by the Ward Food and Nutrition Security Committees, District Food and Nutrition Security Committees and Provincial Food and Nutrition Security Committee. The monitoring and evaluation should be in a tripartite dimension, that is, progress of the interventions, identifying challenges and making necessary recommendations for improvement. The development of the food security implementation model was to a greater extent informed by both Sen’s entitlement and the systems theories. These two theories have common features that work together for the common good as demonstrated in Table 1.

**TABLE 1:** Major characteristics of the entitlement theory and systems theory.

Entitlement theory	Systems theory
Propounded by Sen Armartya	Propounded by Cristina Mele, Jacqueline Pels and Francesco Polese
Looks at the analysis of famines and food (in) security	Looks at system dynamics
The law is a prohibiting factor between food availability and food entitlement	Understanding is realised from the deep appreciation of a whole rather than of individual parts
People affected by famines are not entitled to food but rather to starve	A system is composed of interrelated parts that work together towards the survival of a whole
Legal sources of food: Production based, trade based, own labour and inheritance and transfer entitlements	Boundaries: Demarcations that describe a system and differentiates it from other systems in the surroundings
It is descriptive rather than theoretical	Homeostasis: The ability of a system to endure towards outside variables and uphold its key fundamentals
A general model of analysing famines concentrating on their causation	Adaptation: The ability of a self-regulatory system to effect the inside changes wanted to guard itself and keep satisfying its aim
Theory dwells of food accessibility rather than availability	Cyclical connections that systems connect in such that they control one another
Exchange entitlement failure: Famines are characterised by declines in food access	Feedback loop: A situation by which systems self-rectify themselves based on interactions from other systems in the surroundings

**FIGURE 1:**
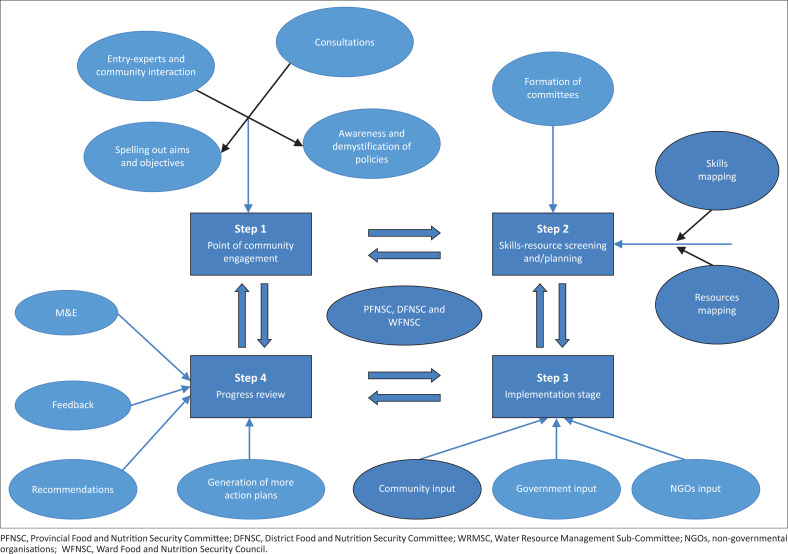
Proposed food security implementation model for Matabeleland South province.

Figure 1 shows the final graphic presentation of the preferred future food security model for enhancing a sustainable food security situation in Matabeleland South Province in Zimbabwe, as designed and constructed by study participants. The structuring of the model followed specific steps at all the levels from village level to the provincial level. Information sharing followed a two-way path at all stages or steps.

## Major characteristics of the entitlement theory and systems theory

A comparison between the two theories used in this research is presented in Table 1.

The development of a sustainable food security implementation model needs a collaborative and a focused effort. To achieve this, the systems theory was relevant in putting together districts and wards together to achieve the four pillars of food security, that is, availability, access, stability and utilisation as propounded by Sen’s entitlement theory.

## The synergistic relationship between entitlement theory and systems theory

Sen (1982:2) described all sources of food as, ‘production-based entitlement, trade-based entitlement, own labour entitlement and inheritance and transfer entitlement’. Gwirayi (2012) defined a system as a coherent unit built up of interconnected and parts that depend on each other that work in unison towards the survival of a whole. Bringing the major focus of the two theories, it can be concluded that the two theories played an imperative role in the study. The entitlement theory looks at food access whilst the systems theory looks at how parts of a system can work together in fighting a common cause. In other words, the entitlement theory spelt out the four legal sources of food but in the community, if parts do not come together in one focus, the community will continue to suffer from food insecurity. The entitlement theory defined the problem of food insecurity but the systems theory informed the affected community of Matabeleland South to bring different interrelated parts to work together in fighting food insecurity.

Table 1 clearly describes the two theories that anchored this study. These theories worked together as shown in Table 1. The Entitlement Theory explored the current trends and realities on food insecurity in general and possible ways that can be used to address the problem. The food security deficit for Matabeleland South Province was approximately 300 000 because of early termination of rains (ZIMVAC 2018). However, the systems theory played a significant role in the development of an implementation model and validating it. The validation was done by stakeholders by validating data generated by the research. These were the same stakeholders who participated in the development of the food security implementation model. The development of a model was done by the people of Matabeleland South Province, and the province can be equated to a system. The province is made up of seven districts that are food insecure. In these districts there are various stakeholders and these can be equated to interrelated parts that work together towards the survival of the whole province.

## Conclusion

The entitlement theory and the systems theory largely inform this study. The theories are combined to reach a common understanding and grounding in developing the food security model for Matabeleland South Province. The research places recognition of food security as an entitlement to human beings, hence the need to develop a model that cuts across issues of availability, access and utilisation in a sustainable and stable way. For food security to be achieved, the view that communities are a system needs to be embraced by using a bottom up approach in developing a food security implementation model. The model has four components, namely point of community engagement, skills-resource screening and or planning, implementation of the model and finally progress review. The operationalisation of this food security implementation model is highly dependent on the communities following the laid down standard operation procedures with clear actions, actors and time with the use of triggers and thresholds for anticipatory action. The development of the food security implementation model contributed in coming up with context-specific food security intervention strategies. The area of developing food security models needs to be thoroughly researched so that context-specific policies can be formulated as indicated by stakeholders. Matabeleland South Province is a system made up of its own people and other resources both artificial and natural and these components are interrelated. This means that it should be taken into cognisance that for any developmental action all the parts of a system should support each other to make a whole, as was the case in developing this food security implementation model for Matabeleland South Province.

## References

[CIT0001] Ashby, J.A., Braun, A.R., Gracia, T., Guerrero, M.P., Hernández, L.A., Quirós, C.A. et al., 2000, *Investing in farmers as researchers: Experience with local agricultural research committees in Latin America*, Centro Internacional deAgricultura Tropical, Cali.

[CIT0002] Bogdanov, A.A., 1922, *Tektology*, Universal Organization Science, Berlin.

[CIT0003] Boulding, K.E., 1956, ‘General systems theory – The skeleton of science’, *Management Science* 2(3), 197–208. 10.1287/mnsc.2.3.197

[CIT0004] Capra, F., 1997, *The web of life: A new scientific understanding of living systems*, Anchor, New York, NY.

[CIT0005] Checkland, P., 1997, *Rhetoric and reality in contracting: Research in and on the NHS*, Oxford University Press, Oxford.

[CIT0006] Edkins, J., 1996, ‘Legality with a vengeance: Famines and humanitarian relief in “complex emergencies”’, *Millennium* 25(3), 547–575. 10.1177/03058298960250030501

[CIT0007] Espejo Villar, L.B., 1999, ‘Hacia un modelo de educación integral: El aprendizaje emocional en la práctica educativa’, *Revista de Ciencias de la Educación* 3(180), 521–535.

[CIT0008] Food and Agricultural Import Regulations, 2019, *Country report*, London, United Kingdom

[CIT0009] Golinelli, P., 2009, *Il Medioevo degli increduli: Miscredenti, beffatori, anticlericali*, University of Naples Federico II, Mursia.

[CIT0010] Gwirayi, P., 2012, ‘An overview of theories on child maltreatment and their applicability to Zimbabwean society’, *Journal of Social Development in Africa* 27(2), 139.

[CIT0011] Jackson, M.C., 2003, *Systems thinking: Creative holism for managers*, p. 378, Wiley, Chichester.

[CIT0012] Luhmann, N., 1990, ‘The cognitive program of constructivism and a reality that remains unknown’, in S. Herting & L. Stein (eds.), *Selforganization*, pp. 64–85, Springer, Dordrecht.

[CIT0013] Meadows, D.H., 2008, *Thinking in systems: A primer*, Chelsea Green Publishing, Chelsea, VT.

[CIT0014] Ng, I.C., Maull, R. & Yip, N., 2009, ‘Outcome-based contracts as a driver for systems thinking and service-dominant logic in service science: Evidence from the defence industry’, *European Management Journal* 27(6), 377–387. 10.1016/j.emj.2009.05.002

[CIT0015] Sen, A., 1982, ‘The right not to be hungry’, in G. Floistad (ed.), *Contemporary Philosophy volume 2*, Martinus Nijhoff, The Hague.

[CIT0016] Sen, A., 1984, ‘Rights and capabilities’, in A. Sen (ed.), *Resources, values and development*, pp. 307–324, Basil Blackwell, Oxford.

[CIT0017] Sen, A.K., 1981, *Poverty and famine: An essay on entitlement and deprivation*, Oxford University Press, Oxford.

[CIT0018] Von Bertalanffy, L., 1956, *General system theory*, General systems, 1, pp. 1–10, Springer, New York, NY.

[CIT0019] Von Bertalanffy, L., 1968, *General system theory*, p. 40, Springer, New York, NY.

[CIT0020] Weinberg, S., 2001, ‘The cosmological constant problems’, in B.A. Weinberg (ed.), *Sources and detection of dark matter and dark energy in the Universe*, pp. 18–26, Springer, Berlin.

[CIT0021] Zimbabwe Vulnerability Assessment Committee (ZIMVAC), 2016, Rural Livelihoods Assessment Report, Harare.

[CIT0022] Zimbabwe Vulnerability Assessment Committee (ZIMVAC), 2018, Zimbabwe Emergency Food Security Assessment Report in collaboration with SADC FAN, Harare.

